# Translation, Adaptation and Psychometric Properties of SATAQ-4R for Brazilian Children

**DOI:** 10.1186/s41155-020-00149-6

**Published:** 2020-07-02

**Authors:** Clara Mockdece Neves, Juliana Fernandes Filgueiras Meireles, Fabiane Frota da Rocha Morgado, Ana Carolina Soares Amaral, Maria Elisa Caputo Ferreira

**Affiliations:** 1 Department of Physical Education, Federal University of Juiz de Fora, Governador Valadares, Brazil; 2 Laboratory of Body’s Study, Federal University of Juiz de Fora, Juiz de Fora, Brazil; 3 Department of Physical Education and Sports, Federal Rural University of Rio de Janeiro, Seropédica, Brazil; 4 Federal Institute of Education, Science and Technology of Southern of Minas Gerais, Barbacena, Brazil; 5 Faculty of Physical Education and Sports, Federal University of Juiz de Fora, Juiz de Fora, Brazil

**Keywords:** Body image, Internalization, Psychometrics, Children

## Abstract

**Background:**

The Sociocultural Attitudes Towards Appearance Questionnaire (SATAQ) is one of the most investigated instruments for the evaluation of sociocultural pressure and internalization of the beauty standard, and it is considered to be one of the most robust instruments for this purpose. The most recent version of this questionnaire is the SATAQ-4R, originally designed for adults and teenagers, which has been used in different countries, contexts, and populations. The cross-cultural adaptation and validation of the SATAQ-4R for Brazilian children are appropriate and necessary.

**Objective:**

The general objective of this study was to translate, adapt, and verify the psychometric qualities of the SATAQ-4R for Brazilian girls and boys aged between 7 and 11 years old.

**Methods:**

Study 1 describes the cross-cultural adaptation, from the translation stage to the pretest in children of both sexes (*n* = 36, *M* = 8.76, SD = 1.59 years). SATAQ-4R has been demonstrated to be easily understood by Brazilian children. Studies 2 and 3 analyze the psychometric qualities of such an instrument in 566 girls (*M* = 9.18, SD = 1.23 years) and in 592 boys (*M* = 9.18, SD = 1.23), respectively. Exploratory and confirmatory factor analyses have been performed with independent samples.

**Results:**

Both instruments presented factor structures composed of five factors, good reliability, and convergent validity.

**Conclusion:**

We concluded that SATAQ-4R-Female and SATAQ-4R-Male are useful tools for evaluating the internalization of beauty standards and of sociocultural pressure on Brazilian children.

## Background

Studies have demonstrated that concerns with the body start in childhood, as children are frequently vulnerable to internalization of appearance ideals and sociocultural pressures (Neves, Cipriani, Meireles, Morgado, & Ferreira, 2017; Leite, Ferrazzi, Mezadri, & Höfelmann, 2014; Spiel, Paxton, & Yager, 2012; Tatangelo, McCabe, Mellor, & Mealey, 2016). Internalization of appearance ideals refers to the degree to which an individual “buys into” socially prescribed appearance ideals, expresses a desire to attain the appearance ideal, and engages in behaviors aimed at meeting those ideals (Thompson, Heinberg, Altabe, & Tantleff-Dunn, 1999; Schaefer, Harriger, Heinberg, Soderberg, & Thompson, 2017). Sociocultural pressures allude to the propagation of this ideal body through different sources of influence, especially parents, friends, and the media, which are considered the most powerful factors for the transmission of the ideal body (Thompson et al., 1999; Schaefer et al., 2017). It is important to underline that the sociocultural influence is considered the main predictor of body dissatisfaction (Amaral & Ferreira, 2017; Cihan, Bozo, Schaefer, & Thompson, 2016; Convertino, Gonzales, Marlcarne, & Blashill, 2019; Rodgers et al., 2011; Schaefer et al., 2017; Stefanile, Nerini, Matera, Schaefer, & Thompson, 2019) that may lead to eating disorders (American Psychiatric Association, 2014).

A qualitative study has shown that Brazilian children from 7 to 11 years old recognized these different sources of sociocultural pressure (Neves, Meireles, Morgado, & Ferreira, 2018). Parents provide the primary context for the formation of body image in children, since individuals have extensive contact with their family during the first years of life (Damiano et al., 2015; Liechty, Clarke, Birky, Harrison, & Kids Team, 2016; Tatangelo et al., 2016). Friends can be considered the second influence factor, as integration into social groups has a decisive impact on body image (Harrison, Rowlinson, & Hill, 2016; Michael et al., 2014; Tatangelo et al., 2016; Tatangelo & Ricciardelli, 2013). Additionally, the media has been noted as the most powerful source of sociocultural influence, constantly exposing viewers to body beauty stereotypes (Boyd & Murnen, 2017; Daniels, Layh, & Porzelius, 2016; Jellinek, Myers, & Keller, 2016; Rice, Prichard, Tiggemann, & Slater, 2016; Tatangelo et al., 2016). Moreover, children’s toys are enabling media factors, taking into account their muscular traits (Boyd & Murnen, 2017; Baghurst, Hollander, Nardella, & Haff, 2006; Pope Jr, Olivardia, Gruber, & Borowiecki, 1999; Smolak, 2011) and the fact that dolls also tend to be represented with lean and slim bodies (Boyd & Murnen, 2017; Jellinek et al., 2016; Rice et al., 2016; Smolak, 2011).

In this regard, several studies have identified the internalization of an ideal to be thin in samples containing children (Brault, Aimé, Bégin, Valois, & Craig, 2015; Bird, Halliwell, Diedrichs, & Harcourt, 2013; Evans, Tovée, Boothroyd, & Drewett, 2013; Ross, Paxton, & Rodgers, 2013). Also, girls with normal BMI or overweight tend to report more pressure to be thin in comparison to underweight girls (Brault et al., 2015) and low-weight boys reported more awareness of the norms of the thinner ideal than other boys (Brault et al., 2015). Therefore, it has been noted that the lean-body ideal reinforced by the different sources of influence potentially affects boys and girls of school age.

The Sociocultural Attitudes Towards Appearance Questionnaire (SATAQ) is considered by the scientific community to be one of the most investigated and most robust instruments for the evaluation of sociocultural pressure and internalization of the beauty standard (López-Guimerá & Sánchez-Carracedo, 2010; Sánchez-Carracedo et al., 2012). This instrument, originally developed by Heinberg, Thompson, and Stormer (1995), has been updated several times (Thompson, van den Berg, Roehrig, Guarda, & Heinberg, 2004; Schaefer et al., 2015) and has had its psychometric qualities tested in many different populations (Calogero, Davis, & Thompson, 2004; Jackson & Chen, 2010; Knauss, Paxton, & Alsaker, 2009; Madanat, Hawks, & Brown, 2006; Markland & Oliver, 2008; Rodgers et al., 2016; Rousseau, Valls, & Chabrol, 2010; Stefanile, Matera, Nerini, & Pisani, 2011; Schaefer et al., 2015; Yamamiya et al., 2016). In the Brazilian context, SATAQ-3 has already been validated for teenagers (Amaral, Conti, Ferreira, & Meireles, 2015) and young adults (Amaral, Cordás, Conti, & Ferreira, 2011; Amaral, Ribeiro, Conti, Ferreira, & Ferreira, 2013; Swami et al., 2011).

The most recent version of this questionnaire is the SATAQ-4R, developed by Schaefer et al. (2017). SATAQ-4R evaluates the internalization of appearance ideals and pressures to achieve the social ideal (derived mainly from media, parents, friends, and close people). The most important contribution of this update is the inclusion of the evaluation of general internalization and attractiveness, as well as the pressure coming from other significant people such as romantic partners, coaches, and teachers (Schaefer et al., 2017). In addition, SATAQ-4R has different versions for men and women, capturing appearance-related pressures and appearance ideal internalization among males and females. The instrument is composed of 31 statements in its female version and 28 statements in its male version, with responses made on a Likert scale containing five options (from 1: Strongly Disagree to 5: Strongly Agree). Both versions have seven subscales: (1) internalization: thin/low body fat; (2) internalization: muscular; (3) internalization: general attractiveness; (4) pressures: family; (5) pressures: media; (6) pressure: peers; and (7) pressure: significant others (Schaefer et al., 2017). Higher scores indicate higher internalization of the body ideals and higher pressure perceived to reach such ideals. The SATAQ-4R has already had its psychometric qualities evaluated for men, women, and teenage girls in the USA (Schaefer et al., 2017), Turkish women (Cihan et al., 2016), Italian women and man (Stefanile et al., 2019), and sexual minority adults in the USA (Convertino et al., 2019).

Due to the lack of valid and reliable measures to evaluate body image dimensions among Brazilian children, specially sociocultural influence, and the recommendation of the adaptation of the SATAQ and its versions for this population (Schaefer et al., 2015; Schaefer et al., 2017), the translation, adaptation, and validation of SATAQ-4R for Brazilian children is appropriate and necessary. Furthermore, it will enable a comprehensive evaluation of sociocultural influence on the body image of the Brazilian public. Therefore, the objective of this study is to translate, adapt, and verify the psychometric qualities of SATAQ-4R for Brazilian boys and girls.

## General methods

This article was divided into three studies with the aim of providing a version of SATAQ-4R for Brazilian children. Study 1 describes the initial process, from its translation until the pretest in children of both sexes. In study 2, EFA (exploratory factor analysis), CFA (confirmatory factor analysis), reliability, and convergent validity of SATAQ-4R-Female were performed for Brazilian girls. Similarly, in study 3, the psychometric qualities of SATAQ-4R-Male were evaluated in a sample of boys. The research has been approved by the Committee for Ethics in Research of the Federal University of Juiz de Fora. Consent forms were signed by the parents or guardians of all children participating in the three studies.

We followed the recommendations regarding the process of cross-cultural adaptation of scales (Beaton, Bombardier, Guillemin, & Ferraz, 2000; Guillemin, Bombardier, & Beaton, 1993; Herdman, Fox-Rushby, & Badia, 1997, 1998; Reichenheim & Moraes, 2007). In addition, we took in account the procedures performed at Schaeffer et al. (2017). All the stages were performed from March 2016 to July 2017.

## Study 1: Translation and cross-cultural adaptation of SATAQ-4R for Brazilian children

The aim of this study was to translate the original version of the SATAQ-4R and to pretest it for Brazilian girls and boys. This process included the following stages: literature review and focus group (stage 1), translation and back-translation (stage 2), experts committee (stage 3), and pretest (stage 4).

The author of the article on the development and validation of the original instrument (Schaefer et al., 2017) was contacted in order to ask for permission to adapt it to Brazilian children. His permission was obtained on November 10, 2015 (Thompson, 2015).

### Stage 1: Literature review and focus group

#### Methods

This stage attested the conceptual equivalence of SATAQ-4R. It explored whether the concept covered by the original instrument would be relevant and pertinent to the new context to which it is being adapted (Reichenheim & Moraes, 2007). The evaluation of conceptual equivalence was verified through literature review and also through conducting focus groups with the target audience. Two previous studies have described this stage (Neves et al., 2017; Neves et al., 2018).

A literature review was conducted in Scopus, MEDLINE, and Virtual Health Library databases with an intersection of the keywords “body image” AND “child.” It has identified 7681 references and, after the exclusions, 33 studies were analyzed. More details can be found in Neves et al. (2017).

A qualitative study performed two focus groups with girls (*n* = 10) and two with boys (*n* = 9), aged between 7 and 11 years old (Neves et al., 2018). All the children were recruited from public schools in Juiz de Fora (a city in the southeast of Brazil), as a matter of convenience. Four researchers participated in the focus groups: two moderators, one observer, and one research assistant. A semi-structured guide had been created previously in order to guide the discussion. In the focus groups, the aim was to discover the opinion of the target audience related to their concerns regarding body appearance, as well as to identify if the different sources of influence of SATAQ-4R (parents, friends, close people, and media) are perceived by the children.

#### Results

Literature review (Neves et al., 2017) demonstrated that children are frequently vulnerable to internalization of appearance ideals and sociocultural pressures. Besides that, the authors pointed out previous studies evaluating the influence of family, friends, and media on children’s body image. The focus groups have shown that Brazilian children presented attitudes that indicate a concern with the body in general and specific aspects, besides behaviors related to body image (Neves et al., 2018). Thus, a literature review and a qualitative study indicated the conceptual equivalence of SATAQ-4R in Brazilian children of both sexes.

### Stage 2: Translation and back-translation

#### Methods

In order to verify the semantic equivalence, the translations of the female (SATAQ-4R-Female) and male (SATAQ-4R-Male) versions were made from its original language (English) into the target language (Portuguese). The evaluation of semantic equivalence involves the ability to transfer meaning from the concepts contained in the original instrument to the version, providing a similar effect on respondents in both cultures (Reichenheim & Moraes, 2007). Two bilingual translators, whose native language is Portuguese, each made independent translations of SATAQ-4R-Female and SATAQ-4R-Male. Their questionnaires were compared and the translators created a synthesis of each version. Then, the syntheses were forwarded to two other translators, whose native language is English and that did not know the original scale, in order to make back-translations into the original language of the instrument.

#### Results

The questionnaire title was translated into Portuguese as “Questionário de Atitudes Socioculturais em Relação à Aparência - 4 Revisado,” with the aim of enabling Brazilians to understand it. However, in order to permit the general scientific community to identify and recognize it, the original abbreviation “SATAQ-4R” has been maintained. The versions produced in this stage are available as an Additional file 1.

### Stage 3: Experts’ committee

#### Methods

All processes (translations, syntheses, and back-translations) were gathered and discussed by a committee of experts composed of a linguist, five methodologists, two translators, and two back-translators. The main role of this committee was to verify all versions of the questionnaire and reach an agreement on any discrepancies. Furthermore, this committee also evaluated the operational equivalence. Operational equivalence refers to a comparison between the aspects of using an instrument in the target and source populations, so that efficiency is similar even if the modus operandi is not the same (Reichenheim & Moraes, 2007). They have also evaluated the items, considering its relevance and appropriate format, the instructions, the manner of implementing the scales, and the answer options.

#### Results

After the translations and back-translations of SATAQ-4R-Female and SATAQ-4R-Male were created, the committee of experts modified the writing of some statements in order to enable comprehension by the target audience. Some terms from their original language that permits more than one translation into Portuguese (such as close people/third party, mates/friends, strong/muscular) were analyzed, and the easiest option to understand was chosen. All these points were evaluated subsequently in the pretest stage.

More importantly, the experts decided to change the response options: instead of the five original alternatives of the Likert scale (*Definitely Disagree*, *Mostly Disagree*, *Neither Agree nor Disagree*, *Mostly Agree*, *Definitely Agree*), they suggested to reduce them to three and presented as symbols (,, ). The majority of public elementary school students of 8 years old or more can be considered as functionally illiterate, with levels of reading and mathematics considered insufficient. The situation is even more critical in specific areas such as the North and Northeast, where more than 30% of students have elementary levels of reading and mathematics (Brasil, 2016). We chose to simplify the Likert scale to three points in order to facilitate the interpretation of the scale by the target population. Furthermore, the child-friendly feature in all the alternatives can be considered an attractive factor. This new format was analyzed by the author of the original questionnaire by email, on May 18, 2016 (Thompson, 2016). He has agreed and considered the modifications relevant.

Moreover, when examining some statements, the experts have concluded that their translations into Portuguese would be identical. In the male version, this was the case with the statements 6 (“I don’t really think much about my appearance”) and 7 (“I don’t think much about how I look”). In the female version, they verified similarities in three parts: among statements 5 (“I think a lot about my appearance”), 9 (“I don’t really think much about my appearance”), and 14 (“I don’t think much about how I look”); between statements 3 (“I want my body to look very thin”) and 11 (“I want my body to look very lean”); and between the statements 8 (“I want my body to look muscular”) and 10 (“I don’t want my body to look muscular”). For this reason, it was decided to keep statement 6 for boys and statements 5, 3, and 8 for girls and to exclude the repeated statements. Therefore, the tested version on the pretest stage was composed of 27 statements for boys and 27 for girls.

### Stage 4: Pretest

#### Methods

During the pretest stage, which is the final adaptation process, 20 children were interviewed, 10 girls and 10 boys, aged between 7 and 11 years old (*M* = 8.03, SD = 1.41). The pretest was conducted in individually interviews which aimed to investigate the children’s reactions to any possible difficulties found while filling in SATAQ-4R, in its female and male versions. Besides that, we also evaluated the children’s opinions about the symbols used as an answer option for them (,, ); their understanding about *body appearance*, the difference between the terms *strong* and *muscular*, and their understanding about *family*, *friends*, *close people*, and *media*. These terms are fundamentals for the full understanding of the questionnaire. After a few modifications, it was necessary to develop and conduct a new pretest. This time, 8 girls and 8 boys (*M* = 9.50, *SD* = 1.77) were interviewed on the second wave.

#### Results

In the first pretest, it was verified that the children understood and approved the response options presented as symbols. Additionally, a majority of the participants demonstrated an understanding of the meaning of *body appearance.* When it comes to understanding the words *strong* and *muscular*, it was observed that the children understand the former in its literal sense; in other words, *strong* as having a lot of physical strength. On the other hand, the term *muscular* has been understood as expected: related to muscular appearance or to the quantity of muscles. We decided to keep the word *muscular* in favor of *strong*. Children have appropriately interpreted the meaning attributed to the words *family*, *friends*, and *media.* When it comes to the description of *close people*, the original instrument includes in its description *teachers*, *coaches*, and *romantic partners.* However, as children are the subjects, the last term was replaced by *neighbors*, considering that children mentioned them during the focus groups and that they can be a relevant source of influence in the Brazilian context.

After the first pretest, some changes were made to the instrument, and in the second pretest, no problems could be identified. Therefore, it was decided that the instrument was ready to be implemented on a larger scale.

### Conclusion

Study 1 concludes that the Brazilian version of SATAQ-4R for boys and girls has attested conceptual, semantic, and operational equivalences. One version for each sex was proposed to have its psychometric properties evaluated on studies 2 and 3.

## Study 2: Psychometric properties of SATAQ-4R-Female for Brazilian girls

Study 2 investigated the factorial structure of the translated/adapted version of the SATAQ-4R-Female for Brazilian girls through EFA and CFA, in order to identify specific factors that describe the construct of interest (Hair Júnior, Black, Babin, Anderson, & Tatham, 2009). Reliability (Cronbach’s alpha) and convergent validity were also evaluated. Reliability is concerned with the homogeneity of the scale items (DeVellis, 2012; Hair Júnior et al., 2009). Convergent validity indicates the extent to which the scale correlates with other theoretically related measures (DeVellis, 2012; Hair Júnior et al., 2009). The recommendations of DeVellis (2012), Hair Júnior et al. (2009), Pasquali (2010), and Malhotra (2012) were followed in this stage.

### Methods

#### Participants

The participants of this stage were recruited from different Brazilian public and private schools, selected as a matter of convenience. All five Brazilian regions (North, Northeast, Central West, Southeast, and South) and 16 cities participated, which enabled sample heterogeneity. Included were children between 7 and 11 years of age (from the second until the fifth year of elementary school) who were regularly enrolled and frequently attending classes at the chosen schools. Six hundred three girls participated in the research, and those whose personal details were incomplete (*n* = 33) were excluded from the analyses. The final sample contained 566 female participants between 7 and 11 years old. Demographic information for each sample is provided in Table 1. The girls’ sample has been divided randomly into two equal proportions (*n* = 283) in order to be used for EFA and CFA.

**Table 1:** Demographic information for Brazilian girls

	**Girls**
*N*	566
Age mean (SD)	9.18 (1.23)
Age range	7–11
*BMI categories (%)*	
Underweight	6.7
Normal weight	66.0
Overweight	14.5
Obese	12.8
*Type of school (%)*	
Private school	26.8
Public school	73.2

*Note: BMI* body mass index

#### Instruments

##### Sociocultural Attitudes Towards Appearance Questionnaire-4Revised (SATAQ-4R-Female)

The female version of SATAQ-4R, obtained from the second pretest, was used in this study. It was composed of 27 statements and three response options ( = *Never* [score 1]);  = *Sometimes* [score 2];  = *Always* [score 3]).

##### Silhouette Scale for Brazilian Children (SSBC)

The SSBC (Kakeshita, Silva, Zanatta, & Almeida, 2009) is the only instrument developed in Brazil that is valid for evaluating body dissatisfaction in children from 7 to 12 years old. The scale contains 11 pictures, as individual cards, that range from the thinnest to the widest silhouette. All children answered the question: “Which of these pictures represents your current body?” and “Which of these pictures represent the body you would like to have?” Body dissatisfaction was calculated by the difference between the real and ideal silhouettes. The larger the discrepancy, the higher the level of dissatisfaction, with positive values indicating a desire for a slimmer body silhouette and negative values indicating the opposite. The validity and reliability indices have been verified by Kakeshita et al. (2009). SSBC may therefore be taken as a measure to verify the convergent validity of SATAQ-4R. A positive correlation between SATAQ-4R and SSBC was expected, indicating that the higher one’s body dissatisfaction, the higher the internalization of sociocultural pressures.

##### Direct questions

The participants were asked two questions for the analysis of convergent validity: “On the whole, are you satisfied with your body?” and “On the whole, do you have body concerns?” The options could range from (1) *Strongly Disagree* to (5) *Strongly Agree.* Both questions were developed by the expert committee. We expected that the body satisfaction question would present a negative correlation with SATAQ-4R, while body concern questions would show a positive correlation with SATAQ-4R.

##### Anthropometric data

Body mass and stature were measured for the calculation of body mass index (BMI). The classifications of BMI (underweight, normal weight, overweight, and obese) followed recommendations based on the age (Onis et al., 2007) and were used in the convergent validity analysis. A positive correlation was expected between BMI and SATAQ-4R.

##### Data collection process

In each school, teachers were responsible for collecting data. In order to reduce intra-researcher interference and standardize data collection, teachers received detailed information about the procedures. The children answered the direct questions of SATAQ-4R-Female individually. As soon as they finished, the girls were led to another room in order to have their anthropometric measurements taken and to complete the SSBC, according to the recommendations of Kakeshita et al. (2009). There was no time limit for them to fill out the questionnaires. The average time took by children to complete all data collection procedures was 50 min.

#### Statistical analysis

##### EFA

We assessed the factor structure of the SATAQ-4R using EFA with principal components factory and Promax rotation. The significant Bartlett’s test of sphericity (*p* < .001) as well as the Kaiser–Meyer–Olkin measure (KMO > .6) was adopted in order to verify the appropriateness of the samples to EFA (Hair Júnior et al., 2009). The number of factors was determined by taking into account statistics (eigenvalue > 1.0), analysis of the scree plot, parallel analysis, and theoretical framework. The factor loadings of each statement were verified, and the values above 0.5 were considered appropriate (Hair Júnior et al., 2009). This analysis was performed on SPSS v.21.0.

##### CFA

CFA was applied in order to confirm the factor structure identified in EFA. In order to be considered suitable, this model must present the following parameters: root mean square error (RMSEA) < .08; *χ*^2^/GL < 3; Goodness of Fit Index (GFI), Parsimony Goodness of Fit Index (AGFI), Normed Fit Index (NFI), Non-Normed Fit Index (NNFI), and Comparative Fit Index (CFI) > .9 (Hair Júnior et al., 2009). CFA was carried out on LISREL v.8.51.

##### Reliability

With the aim of testing reliability, the internal consistency of each factor of the scale was evaluated through Cronbach’s alpha (Hair Júnior et al., 2009). Values above .60 are considered satisfactory, and those above .80 are excellent (Hair Júnior et al., 2009).

##### Convergent validity

Convergent validity was assessed using Spearman’s correlations between the SATAQ-4R-Female subscales and the other constructs of interest including body dissatisfaction measurements (SSBC), body satisfaction and concerns (direct questions), and BMI. Evans et al. (2013) developed a theoretical model for children which considers thin-ideal internalization as related to body dissatisfaction and BMI. Based on these findings, we expected a positive correlation between SATAQ-4R subscales and BMI, body dissatisfaction (SSBC), and body concern (directed question) and negative correlation between SATAQ-4R subscales and body satisfaction (directed question). Correlations of .20, .40, and .60 were considered weak, moderate, and strong, respectively (Hair Júnior et al., 2009).

### Results

#### Exploratory factor analysis

Bartlett’s test of sphericity was significant (*χ*^2^ = 3035.657, *gl* = 231; *p* < .0001) and the value of Kaiser–Meyer–Olkin was .899, which suggests that the statements were adequate to factor analysis. Five statements were deleted (“It is important for me to look good in the clothes I wear.” “I think a lot about my appearance.” “I want to be good looking.” “It is important to me to be attractive.” “Family members encourage me to get in better shape.”) due to not have reached sufficient values in EFA indicators (e.g., communalities, individual item KMO, or factorial load). Eigenvalue, scree plot, and parallel analysis showed a solution of five factors (*internalization: muscular; internalization: thin/low body fat; internalization: ideal appearance; pressures: peers/SO; pressures: media*), instead of seven, as in its original version for American women (*internalization: thin/low body fat; internalization: muscular; internalization: general attractiveness; pressures: family; pressures: peers; pressures: significant others; pressures: media)* (Schaefer et al., 2017). On the subscales *internalization: muscular* and *pressures: media*, the statements were kept in their primary factors with high factor loading (> 0.692). The subscale *internalization: thin/low body fat* included three other statements from this subscale and two statements from *pressures: family* that led to the lean body standard. All statements related to improving the appearance (13, 17, 21, and 22) were joined into one single factor, renamed as *internalization: ideal appearance.* The rest of the statements on the subscales *pressure: peers* and *pressure: significant others* were assembled into *pressure: peers/significant others.* Table 2 presents EFA indicators from the solution of five factors for SATAQ-4R-Female.

**Table 2:** EFA indicators from the solution of five factors for SATAQ-4R-Female

	**Internalization**			**Pressures**	
	**Muscular**	**Thin/low body fat**	**Ideal appearance**	**Peers/SO**	**Media**
Eigenvalues	1.385	8.045	1.050	2.220	1.635
% Variance	6.296	36.570	4.773	10.089	7.433
Cronbach’s alpha	.842	.818	.743	.834	.846

*Note: EFA* exploratory factor analysis, *SATAQ-4R* Sociocultural Attitudes Towards Appearance Questionnaire-4Revised, *SO* significant others

#### Confirmatory factor analysis

**Fig. 1:**
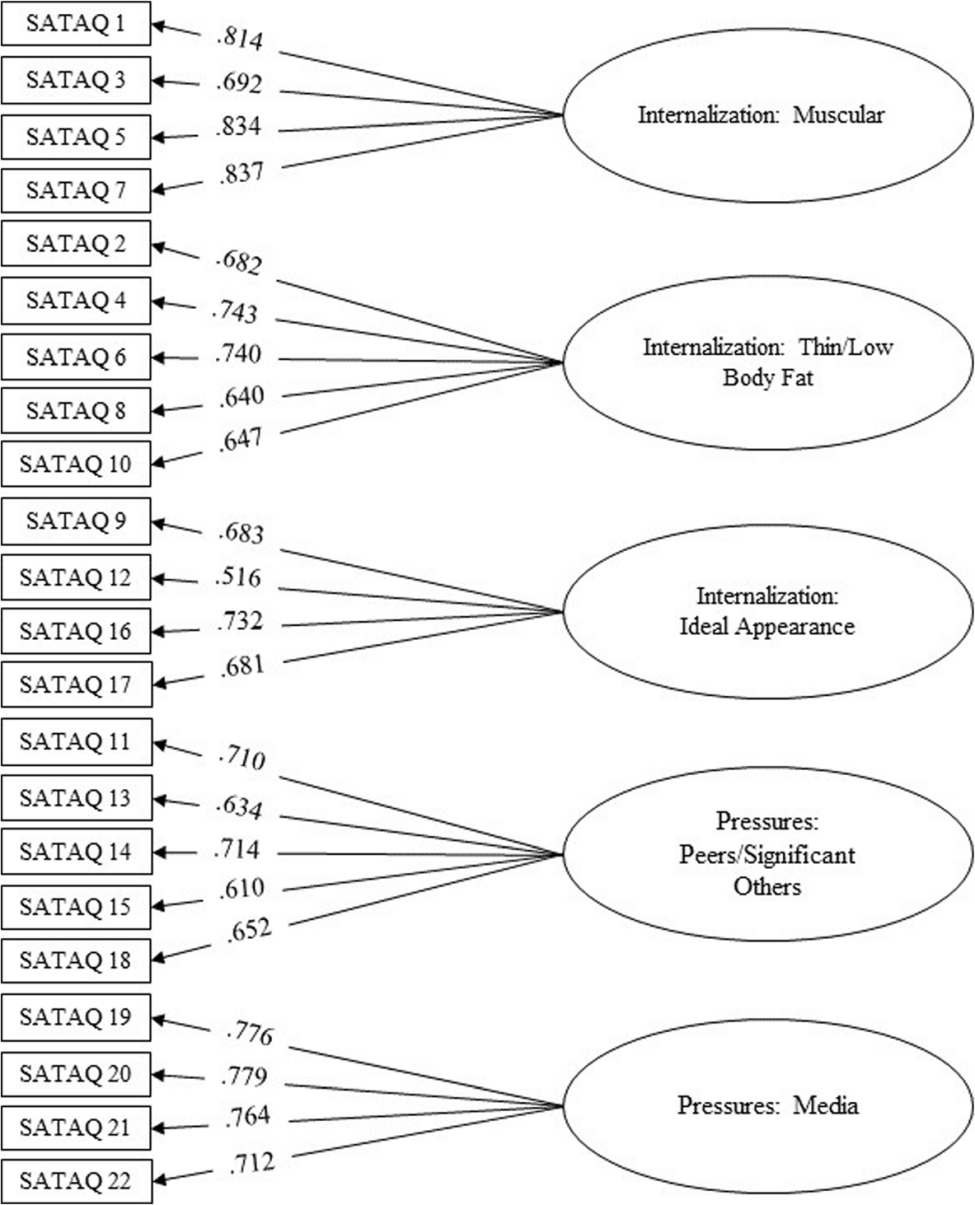
Factor structure of the SATAQ-4R for Brazilian girls

CFA examined the factor structure proposed in EFA. Satisfactory adjustment of five factors and 22 statements were identified for the model. Adjustment indicators of the SATAQ-4R for Brazilian girls are presented in Table 3. The factor loadings of the statements in their respective factors were high, varying from 0.575 to 0.814. Therefore, this factor structure may be considered adequate, given that all figures have been judged as suitable. Figure 1 shows the factor structure of the SATAQ-4R-Female.

**Table 3:** Adjustment indicators of the SATAQ-4R for Brazilian girls

**Adjustment indicators**	**Reference values**^**a**^	**SATAQ-4R values**
Qui-quadrado/degrees of freedom (*χ*^2^/*df*)	Ideal < 3	2.50
Root-mean-square error of approximation *(RMSEA)*	< .08	.073
Goodness of Fit Index (GFI)	> .90	.982
Adjusted Goodness of Fit Index (AGFI)	> .90	.978
Normed Fit Index (NFI)	> .90	.976
Non-Normed Fit Index (NNFI)	> .90	1.000
Comparative Fit Index (CFI)	> .90	1.000

^a^Hair Júnior et al. (2009)

#### Reliability

Cronbach’s alpha for each subscale is shown in Table 2. The internal consistency of SATAQ-4R-Female subscales ranged between of .743 and .846, which is considered adequate (Hair Júnior et al., 2009).

#### Convergent validity

Table 4 shows the correlation between SATAQ-4R-Female subscales. The correlations between the subscales have presented weak or moderate intensity.

**Table 4:** Intercorrelations between SATAQ-4R-Female subscales, body satisfaction, body concern, BMI, and SSBC in Brazilian girls

	**Mean (SD)**	**1**	**2**	**3**	**4**	**5**
**1- Internalization: muscular**	5.79 (2.39)					
**2- Internalization: thin/low body fat**	7.79 (3.00)	.362*				
**3- Internalization: ideal appearance**	5.72 (2.18)	.382*	.451*			
**4- Pressures: peers/significant others**	6.43 (2.41)	.363*	.560*	.668*		
**5- Pressures: media**	5.68 (2.41)	.308*	.421*	.515*	.518*	
**Body satisfaction**	4.07 (1.20)	− .049	− .194*	− .122*	− .113*	− .173*
**Body concern**	3.70 (1.45)	.128*	.271*	.191*	.177*	.148*
**BMI**	18.07 (3.70)	.129*	.398*	.143*	.262*	.208*
**SSBC**	0.97 (2.48)	.054	.411*	.076	.241*	.121*

*Note: SATAQ-4R* Sociocultural Attitudes Towards Appearance Questionnaire-4Revised, *BMI* body mass index, *SSBC* Silhouette Scale for Brazilian Children

*p < 0.01

Additionally, Table 4 also demonstrates the correlations between SATAQ-4R-Female subscales, with the convergent measures (body satisfaction, body concern, BMI, and silhouette scales). Body satisfaction, evaluated through direct questions, has been associated significantly and negatively with SATAQ-4R-Female subscales, except with *internalization: muscular*. Body concern and BMI have been correlated positively with SATAQ-4R-Female five subscales. Body dissatisfaction, evaluated through SSBC, has been positively correlated to SATAQ-4R-Female, *internalization: thin/low body fat*, *pressures: peers/significant others,* and *pressures: media.*

## Study 3: Psychometric properties of SATAQ-4R-Male for Brazilian boys

The aim of this study was to evaluate the factorial structure of SATAQ-4R-Male in Brazilian boys, as well as to analyze the convergent validity and reliability in this sample. The methodological procedures, including instruments, processes, and data analysis were the same as described in study 2 (DeVellis, 2012; Hair Júnior et al., 2009; Pasquali, 2010; Malhotra, 2012).

### Methods

#### Participants

The participants in this stage were recruited from the same schools as study 2. Six hundred thirty-five boys took part, and those whose details were incomplete (*n = 43*) were excluded from the analysis. The final sample had 592 male participants, between 7 and 11 years old. See Table 5 for sample sociodemographic information. EFA and CFA were carried out with two male’s independent samples which were randomly chosen (*n = 269*).

**Table 5:** Demographic information for Brazilian boys

	**Boys**
*N*	592
Age mean (SD)	9.32 (1.24)
Age range	7–11
*BMI categories (%)*	
Underweight	7.9
Normal weight	62.0
Overweight	11.2
Obese	18.9
*Type of School (%)*	
Private school	27.8
Public school	72.2

*Note: BMI* body mass index

### Results

#### Exploratory factor analysis

Based on Bartlett’s test of sphericity (*χ*^2^ = 3846.243, *gl* = 276; *p* < .0001) and on the value achieved on Kaiser–Meyer–Olkin (.918), the statements of SATAQ-4R on its male version were considered appropriate for the factor analysis.

Three statements (“I feel pressure from family members to look thinner,” “I feel pressure from my peers to look in better shape,” and “I feel pressure from family members to improve my appearance”) were excluded due to not have reached sufficient values in EFA indicators (e.g., communalities, individual item KMO, or factorial load). Eigenvalue, screen plot, and parallel analysis have demonstrated a solution of 25 statements, divided into five factors, as presented in Table 6. Instead of the original seven factors *(internalization: thin/low body fat; internalization: muscular; internalization: general attractiveness; pressures: family; pressures: peers; pressures: significant others; pressures: media)*, just as in the female version, the factors received the following names: *internalization: muscular*, *internalization: thin/low body fat*, *internalization: ideal appearance*, *pressures: family/peers/significant others* and *pressures: media.*

**Table 6:** EFA indicators from the solution of five factors for SATAQ-4R-Male

	**Internalization**			**Pressures**	
	**Muscular**	**Thin/low body fat**	**Ideal appearance**	**Family/peers/SO**	**Media**
Eigenvalues	1.551	1.311	1.110	9.484	2.074
% Variance	6.643	5.464	4.626	39.515	8.643
Cronbach’s alpha	.861	.690	.612	.893	.863

*Note: EFA* exploratory factor analysis, *SATAQ-4R* Sociocultural Attitudes Towards Appearance Questionnaire-4Revised, *SO* significant others

#### Confirmatory factor analysis

**Fig. 2:**
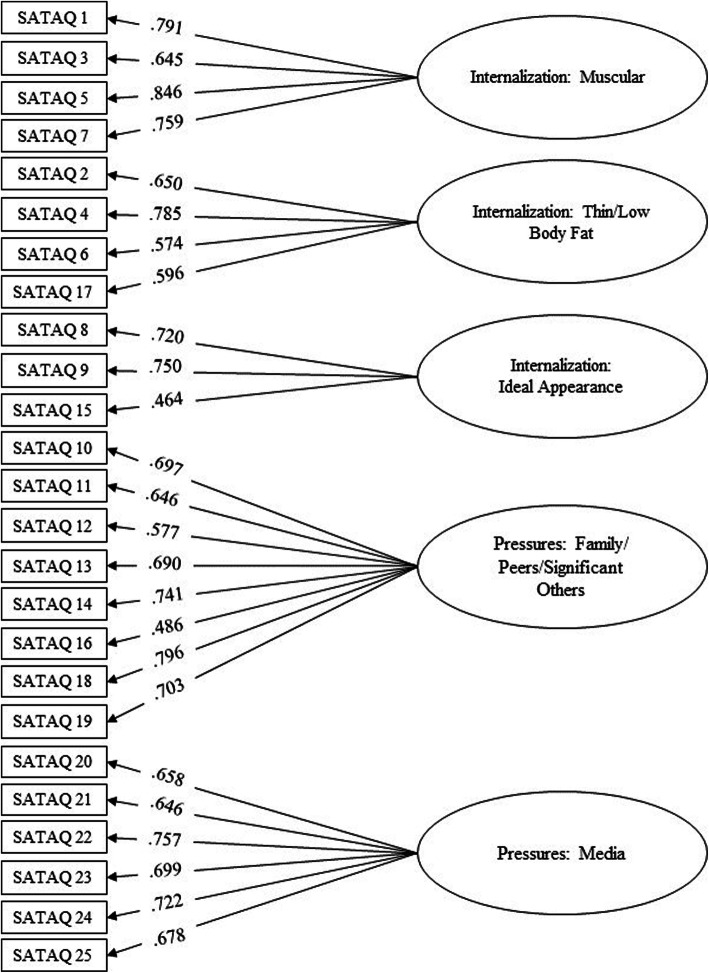
Factor structure of the SATAQ-4R for Brazilian boys

CFA indicated that having 5 factors composed of 25 statements has enabled adequacy in the data. Adjustment indicators of the SATAQ-4R for Brazilian boys are presented in Table 7. The factor loading of the statements in their respective factors was high, varying from 0.435 to 0.840. Figure 2 shows the factor structure of the SATAQ-4R-Male.

**Table 7:** Adjustment indicators of the SATAQ-4R for Brazilian boys

**Adjustment indicators**	**Reference values**^**a**^	**SATAQ-4R values**
Qui-quadrado/degrees of freedom (*χ*^2^/*df*)	Ideal < 3	2.87
Root-mean-square error of approximation *(RMSEA)*	< .08	.079
Goodness of Fit Index (GFI)	> .90	.970
Adjusted Goodness of Fit Index (AGFI)	> .90	.963
Normed Fit Index (NFI)	> .90	.959
Non-Normed Fit Index (NNFI)	> .90	.985
Comparative Fit Index (CFI)	> .90	.987

^a^Hair Júnior et al. (2009)

#### Reliability

Cronbach’s alpha of each subscale is presented in Table 6. The internal consistency of SATAQ-4R-Male subscales ranged between of .612 and .893, which is considered satisfactory (Hair Júnior et al., 2009).

#### Convergent validity

The correlations among the SATAQ-4R male subscales were moderate, which can be observed in Table 8.

**Table 8:** Intercorrelations between SATAQ-4R-Male subscales, body satisfaction, body concern, BMI, and SSBC in Brazilian boys

	**Mean (SD)**	**1**	**2**	**3**	**4**	**5**
**1- Internalization: muscular**	8.47 (2.86)					
**2- Internalization: thin/low body fat**	6.34 (2.24)	.399*				
**3- Internalization: ideal appearance**	5.37 (1.92)	.280*	.514*			
**4- Pressures: family/peers/significant Others**	12.98 (5.09)	.573*	.537*	.584*		
**5- Pressures: media**	9.05 (3.65)	.404*	.457*	.429*	.614*	
**Body satisfaction**	4.00 (1.28)	− .067	− .158*	− .097*	− .087	− .041
**Body concern**	3.22 (1.55)	.153*	.221*	.147*	.144*	.141*
**BMI**	18.22 (3.73)	− .079	.204*	.151*	− .021	.013
**SSBC**	0.29 (2.39)	.082*	.166*	.063	− .062	.016

*Note: SATAQ-4R* Sociocultural Attitudes Towards Appearance Questionnaire-4Revised, *BMI* body mass index, *SSBC* Silhouette Scale for Brazilian Children

*p < 0.01

Body satisfaction has been associated negatively with *internalization: thin/low body fat* and *internalization: ideal appearance*. A positive correlation has been found among the questions regarding body concern and SATAQ-4R-Male five subscales. The results related to BMI point to a positive correlation between *internalization: thin/low body fat* and *internalization: ideal appearance*. The body dissatisfaction evaluated by SSBC correlated positively with *internalization: muscular* and *internalization: thin/low body fat*.

## General discussion

This article aimed to adapt SATAQ-4R for Brazilian children, as well as to verify their psychometric qualities in girls and boys. Study 1 produced a cross-cultural adaptation of SATAQ-4R, taking into account the particular needs of children. The literature review notes that different versions of SATAQ have been already used with children (Neves et al., 2017). Smolak, Levine, and Thompson (2001) have validated SATAQ-1 with 14 statements and five answer options on a Likert scale for middle-school boys and girls. The authors have changed some of the original questions in order to enable their understanding, such as using positive sentences and renaming magazine titles for a child context. Evans et al. (2013), Bird et al. (2013), and Ross et al. (2013) have applied this instrument with a reduced number of statements, taking into account the peculiarities of this sample. Thus, adaptations have been made in order to enable a better understanding of this instrument by children.

Furthermore, the focus groups verified the applicability of SATAQ-4R for Brazilian children of both sexes. After the translations and back-translations, the committee of experts indicated the need to reduce the number of response options from five to three in order to provide clarity to the language of some statements, making the instrument appropriate for the target age. Mellor, Moore, and K. A. (2014) argue that the number of Likert scale response options in children’s questionnaire has not been well established, varying from 3 to 5 options. Jacoby and Matel (1971) and Pasquali (2010) recommended the use of 3-point Likert scale for children in order to facilitate their interpretation of the answers. Johns (2010) explained that a 3-point Likert scale shows the direction of response rather than the strength of opinion. In children, the 3-Likert scale has been previously used to evaluate different constructs (Clance, Mitchell, & Engelman, 1980; Goodman, 1997; Kovacs, 1992; Wolfe, 1996).

The pretest confirmed the children’s understanding regarding the statements and response options. Therefore, the Brazilian versions of SATAQ-4R-Female and SATAQ-4R-Male have presented conceptual equivalence on their statements, as well as the semantic and operational equivalence of statements, and may be considered adequate for the test and its psychometric properties.

Study 2 aimed to verify the factor structure of SATAQ-4R-Female through EFA and CFA in girls aged from 7 to 11 years old. The best fit model was composed of five factors and 22 statements, which was similar to the original version for American women (Schaefer et al., 2017). Although a reduction may have occurred in the number of subscales (from seven to five), it was observed that the factors kept in this study had similarities with the original validity study. The subscales *internalization: thin/low body fat, internalization: muscular* and *pressures: media* have remained very close to the original. Just like American adults (Schaefer et al., 2017), Brazilian children recognize the internalization of the ideal of a slim and muscular body, as well as the pressure applied by the media in order to achieve a beauty stereotype.

The subscale *internalization: ideal appearance* included statements (items 9, 12, 16, and 17) that originally belonged to *pressures: family, pressures: peers* and *pressures: significant others*. They were worded around improving the appearance or getting in a better shape. Based on the statistics, the purpose of the pressure was stronger than the influence itself for Brazilian girls. It seems that children have more easily recognized the goal of the pressure than who were responsible for them (family, friends, or neighbors). This set of items is different than the other versions of SATAQ-4R (Cihan et al., 2016; Schaefer et al., 2017). Possibly it has happened due to the sample characteristics. Cihan et al. (2016) have evaluated Turkish women and Schaefer et al. (2017) have examined college-age men and women, as well as adolescent girls. It is recommended that future researchers be careful when using it with children.

On the Brazilian girls’ sample, the subscale *pressures: peers/significant others* included statements that, with American college women, belonged to two different factors: *pressure: peers* and *pressures: significant others* (Cihan et al., 2016; Schaefer et al., 2017). The same has happened with both versions of SATAQ-4R for Turkish women (Cihan et al., 2017) and American teenagers (Schaefer et al., 2017). Cihan et al. (2017) justify this difference because of the Turkish language and culture, in which *peers* are frequently considered a subgroup of *significant others* in somebody’s life. Schaefer et al. (2017) explain that this adjustment reflects the differences in cognitive development of age between college students and teenagers, in order to distinguish both social groups. Both justifications may apply to the Brazilian context. Consequently, friends and close people may not represent totally different groups for the evaluated sample.

In study 3, the factorial structure of SATAQ-4R-Male for boys between 7 and 11 years old was analyzed through EFA and CFA. The best fit model has been identified to be composed of 25 statements and five factors, which were renamed as the following: *internalization: muscular, internalization: thin/low body fat, internalization: ideal appearance, pressures: family/peers/significant others,* and *pressures: media*. Therefore, the factor structure found for boys was consistent with that for girls. Corroborating these results, Schaefer et al. (2017) have also verified similar factor structures on samples of male and female college students. According to the authors, the main difference between both versions was the inclusion of statements which contained the pressures perceived by males regarding muscularity. In this study, SATAQ-4R-Male for Brazilian boys was different from the girls, when it comes to the factor *pressures: family/peers/significant others*. This factor included the statements related to the pressure from friends and close people, as well as two statements related to family influence.

When it comes to convergent validity, it was verified that the SATAQ-4R-Female subscales and SATAQ-4R-Male subscales presented relevant positive correlations regarding body concern and negative correlations regarding body satisfaction. These results have supported the initial hypothesis, considering that the higher the internalization of beauty standards and sociocultural pressures to achieve such models, the higher the body concern and the lower the body satisfaction. Similar results have been obtained with American teenagers and college students (Schaefer et al., 2017), as well as with Turkish women (Cihan et al., 2016).

Additionally, BMI and body dissatisfaction in girls, evaluated through SSBC, correlated positively with SATAQ-4R-Female. Similarly, Evans et al. (2013) have demonstrated that SATAQ-1 has maintained correlation with BMI and a scale of silhouettes, in British girls from 7 to 11 years old. On the male sample of this study, BMI was correlated with *internalization: thin/low body fat* and *internalization: ideal appearance*. SSBC has been associated with *internalization: muscular* and *internalization: thin/low body fat.* The comparison of these results for males is more difficult due to the lack of studies about them. Future studies are recommended in order to enable a better understanding of these relations. Therefore, the convergent validity of SATAQ-4R-Female and of SATAQ-4R-Male is based on BMI, body satisfaction, body concern, and silhouette scales.

### Limitations

This article presents important contributions despite its limitations. First, the fact that data collection has been carried out by different people should be considered a limitation. This aspect contributes to the increase of intra-researcher interference. Nonetheless, this was a required measure to ensure sample heterogeneity in this study, including children from all five regions, given Brazil’s large geographic area. Moreover, detailed instructions were provided and explained to all parties, in order to standardize this process.

Second, there is a lack of instruments valid for Brazilian children, which makes it difficult to evaluate convergent validity. In this study, only the Silhouette Scale for Brazilian Children (Kakeshita et al., 2009) was used to evaluate body dissatisfaction. In order to help evaluate convergent validity, two direct questions about body concern and body satisfaction were also used. The three measures have demonstrated signs of convergent validity with SATAQ-4R for children, as expected.

Third, the number of items and the 3-Likert scale response has to be considered to compare different samples globally. The cross-cultural adaptation process may include changes in the number of items. Schaefer et al. (2017), for example, validated SATAQ-4R with 28 items for men and 31 for women. The Brazilian versions for children are composed of 22 items for girls and 25 items for boys. For future comparisons, statistical resources are needed. Regarding the response options, the validated version in the present research has three options of answers, which took into account the specific target population. Children aged 7 to 11 years, with three response options, had less doubts. In addition, the symbols contributed to the interest of the participants as well as to their understanding. Other versions of SATAQ validated for adolescents and adults have five response options on a Likert-type scale. Comparisons are possible through the use of statistical strategies. We believe that both versions of SATAQ-4R for Brazilian children considered the particularities of the target population.

### Implications

According to Schaeffer et al. (2017), the SATAQ-4R improves upon the previous version of the scale in several important ways, which impacts in its implications. First, SATAQ-4R contributes to the evaluation of the internalization of appearance ideals and pressures to achieve the social ideal among females and males. Second, SATAQ-4R allows for a more precise assessment of sociocultural influences as a factor in understanding body image and eating disturbances since childhood. Third, SATAQ-4R may be useful as an instrument to gauge the merits of body image and eating concerns interventions. Fourth, SATAQ-4R might be applied in order to test theoretical models of the etiology of disordered eating, such as the tripartite influence model.

## Conclusion

SATAQ-4R has been translated and adapted cross-culturally to Brazil and has presented psychometric qualities for Brazilian girls and boys. All the evaluated parameters have achieved recommended values, confirming the validity of models for both sexes. Therefore, the results have indicated that SATAQ-4R-Female and SATAQ-4R-Male are valid and reliable measures for evaluating the internalization of social standards of beauty (*thin/low body fat, muscular*, and *ideal appearance*) and the influence of sociocultural pressure (family, peers, significant others, and media) in boys and girls, respectively. SATAQ-4R has presented five factors, and its subscales have exhibited good reliability and convergent validity. The results of the three studies support the utility of this scale as a new evaluation tool for children in Brazil (see final versions at Additional file 2). The usage of these instruments for both populations is recommended. Additionally, it is suggested that future studies be done to evaluate the psychometric properties of SATAQ-4R in Brazilian teenagers and adults.

## Supplementary information

**Additional file 1: Table S1.** Semantical Equivalence of SATAQ-4R for Brazilian girls. Table S2. Semantical Equivalence of SATAQ-4R for Brazilian boys.

**Additional file 2.** Questionário de Atitudes Socioculturais em Relação à Aparência – 4 R – MASCULINA and Questionário de Atitudes Socioculturais em Relação à Aparência – 4 R – FEMININA.

## Data Availability

Data can be requested to the first author by the e-mail address listed in the contact details.

## Abbreviations

AGFI: Parsimony Goodness of Fit Index
BMI: Body mass index
CFA: Confirmatory factor analysis
CFI: Comparative Fit Index
EFA: Exploratory factor analysis
GFI: Goodness of Fit Index
KMO: Kaiser–Meyer–Olkin measure
NFI: Normed Fit Index
NNFI: Non-Normed Fit Index
RMSEA: Root mean square error
SATAQ: Sociocultural Attitudes Towards Appearance Questionnaire
SATAQ-4R: Sociocultural Attitudes Towards Appearance Questionnaire-4 Revised
SO: Significant others
SSBC: Silhouette Scale for Brazilian Children
