# Supplementary material for: Translation, Adaptation and Psychometric Properties of SATAQ-4R for Brazilian Children
Source: Psicol Reflex Crit. 2020 Jul 2;33:12. doi: 10.1186/s41155-020-00149-6 (PMC7332665; doi:10.1186/s41155-020-00149-6)
Supplement: Supplementary file 1 — Additional file 1: Table S1. Semantical Equivalence of SATAQ-4R for Brazilian girls. Table S2. Semantical Equivalence of SATAQ-4R for Brazilian boys. [file 41155_2020_149_MOESM1_ESM.docx]

***Appendix A***

***Semantical Equivalence of SATAQ-4R for Brazilian girls***

| **ORIGINAL** | **SÍNTESE** | **RETROTRADUÇÃO 1** | **RETROTRADUÇÃO 2** | **PORTUGUESE VERSION** |
| --- | --- | --- | --- | --- |
| It is important for me to look muscular. | É importante para mim parecer musculosa. | It is important to me to appear muscular. | It is important to me to look muscular. | Para mim, é importante parecer musculosa. |
| It is important for me to look good in the clothes I wear. | É importante para mim parecer bem nas roupas que eu uso. | It is important to me to look nice in the clothes I wear. | It is important to me to look good in the clothes that I wear. | ***^b^*** |
| I want my body to look very thin. | Eu quero que meu corpo se pareça muito magro. | I want my body to appear very skinny. | I want my body to be thinner. | Eu quero que meu corpo pareça muito magro. |
| I think a lot about looking muscular. | Eu penso muito sobre parecer musculosa. | I think a lot about appearing muscular. | I think about looking muscular a lot. | Eu gasto muito tempo pensando em ser musculosa. |
| I think a lot about my appearance. | Eu penso muito sobre o meu corpo. | I think a lot about my appearance. | I think about my appearance a lot. | ***^b^*** |
| I think a lot about looking thin. | Eu penso muito sobre parecer magra. | I think a lot about appearing skinny. | I think about being thin a lot. | Eu gasto muito tempo pensando em ser magra. |
| I want to be good looking. | Eu quero estar bonita. | I want to have a good appearance. | I want to have a good appearance. | ***^b^*** |
| I want my body to look muscular. | Eu quero que meu corpo se pareça musculoso. | I want my body to appear muscular. | I want my body to look muscular. | Eu gostaria de ter um corpo muito musculoso. |
| I don’t really think much about my appearance. | Eu realmente não penso muito sobre minha aparência. | I really do not think about my appearance very much. | I honestly don't think much about my appearance. | ***^a^*** |
| I don’t want my body to look muscular. | Eu não quero que meu corpo se pareça forte. | I do not want my body to appear muscular. | I don't want my body to look muscular. | ***^a^*** |
| I want my body to look very lean. | Eu quero que meu corpo se pareça muito magro. | I want my body to appear very slender. | I want my body to look very thin. | ***^a^*** |
| It is important to me to be attractive. | È importante para mim ser bonita. | Being attractive is important to me. | It is important to me to be attractive. | ***^b^*** |
| I think a lot about having very little body fat. | Eu penso muito sobre ter muito pouca gordura no meu corpo. | I think a lot about having very little body fat. | I think about having a low percentage of body fat a lot. | Eu gasto muito tempo pensando em emagrecer. |
| I don’t think much about how I look. | Eu não penso muito sobre como eu me pareço. | I do not think much about how I appear. | I don't think much about my looks. | ***^a^*** |
| I would like to have a body that looks very muscular. | Eu gostaria de ter um corpo muito musculoso. | I would like to have a very muscular body. | I would like to have a very muscular body. | Eu quero que meu corpo pareça musculoso. |
| I feel pressure from family members to look thinner. | Sinto-me pressionada por meus familiares a parecer mais magra. | I feel pressured by my family members to appear skinnier. | I feel pressured by my family to look thinner. | Sinto que minha família me pressiona a ser mais magra. |
| I feel pressure from family members to improve my appearance. | Sinto-me pressionada por meus familiares a melhorar a minha aparência. | I feel pressured by my family members to improve my appearance. | I feel pressured by my family to improve my appearance. | Sinto que minha família me pressiona a melhorar minha aparência. |
| Family members encouraged me to decrease my level of body fat. | Meus pais falam para eu diminuir a gordura do meu corpo. | My family members encourage me to lower my body fat level. | My family encourages me to lower my percentage of body fat. | Minha família me incentiva a emagrecer. |
| Family members encourage me to get in better shape. | Meus familiares me encorajam a chegar a uma melhor forma. | My family members encourage me to get in better shape. | My family encourages me to get in better shape. | ***^b^*** |
| My peers encourage me to get thinner. | Meus amigos falam para que eu fique mais magra. | My colleagues encourage me to stay skinnier | My colleagues encourage me to be thinner. | Meus amigos me incentivam a ficar mais magra. |
| I feel pressure from my peers to improve my appearance. | Sinto-me pressionada por meus colegas a melhorar minha aparência. | I feel pressured by my colleagues to improve my appearance. | I feel pressured by my colleagues to improve my appearance. | Sinto que meus amigos me pressionam a melhorar minha aparência. |
| I feel pressure from my peers to look in better shape. | Sinto-me pressionada por meus colegas a estar em uma melhor forma. | I feel pressured by my colleagues to be in better shape. | I feel pressured by my colleagues to be in better shape. | Sinto que meus amigos me pressionam a melhorar minha forma física. |
| I get pressure from my peers to decrease my level of body fat. | Meus amigos falam para eu diminuir a gordura do meu corpo. | I feel pressured by my colleagues to lower my body fat level. | I feel pressured by my colleagues to decrease my percentage of body fat. | Meus amigos me pressionam a emagrecer. |
| Significant others encourage me to get thinner. | Pessoas próximas falam para eu ficar mais magra. | The people close to me encourage me to stay skinner. | People around me encourage me to be thinner. | Pessoas próximas me incentivam a ficar mais magra. |
| I feel pressure from significant others to improve my appearance. | Sinto-me pressionada por pessoas próximas a melhorar minha aparência. | I feel pressured by the people close to me to improve my appearance. | I feel pressured by people around me to improve my appearance. | Sinto que pessoas próximas me pressionam a melhorar minha aparência. |
| I feel pressure from significant others to look in better shape. | Sinto-me pressionada por pessoas próximas a estar em uma melhor forma. | I feel pressured by the people close to me to be in better shape. | I feel pressured by people around me to be in better shape. | Sinto que pessoas próximas me pressionam a melhorar minha forma física. |
| I get pressure from significant others to decrease my level of body fat. | Sinto-me pressionada por pessoas próximas a diminuir meu nível de gordura corporal. | I feel pressured by the people close to me to lower my body fat level | I feel pressured by people around me to lower my percentage of body fat. | Sinto que pessoas próximas me pressionam a emagrecer. |
| I feel pressure from the media to look in better shape. | Sinto-me pressionada pela mídia a estar em uma melhor forma. | I feel pressured by the media to be in better shape. | I feel pressured by the media to be in better shape. | Sinto que a TV e a Internet me pressionam a melhorar minha forma física. |
| I feel pressure from the media to look thinner. | Sinto-me pressionada pela mídia a estar mais magra. | I feel pressured by the media to be skinnier. | I feel pressured by the media to be thinner. | Sinto que a TV e a Internet me pressionam a parecer mais magra. |
| I feel pressure from the media to improve my appearance. | Sinto-me pressionada pela mídia a melhorar minha aparência. | I feel pressured by the media to improve my appearance. | I feel pressured by the media to improve my appearance. | Sinto que a TV e a Internet me pressionam a melhorar minha aparência. |
| I feel pressure from the media to decrease my level of body fat. | Sinto-me pressionada pela mídia a diminuir meu nível de gordura corporal. | I feel pressured by the media to lower my body fat level. | I feel pressured by the media to decrease my percentage of body fat. | Sinto que a TV e a Internet me pressionam a emagrecer. |

***Note: ^a^=Item deleted at Stage 3 (experts committee); ^b^=Item deleted at EFA***

***Semantical Equivalence of SATAQ-4R for Brazilian boys***

| **ORIGINAL** | **TRANSLATION** | **BACKTRANSLATION 1** | **BACKTRANSLATION 2** | **PORTUGUESE VERSION** |
| --- | --- | --- | --- | --- |
| It is important for me to look muscular. | É importante para mim parecer forte. | It is important to me to seem masculine. | It is important for me to look muscular. | Para mim, é importante parecer musculoso. |
| I want my body to look very thin. | Eu quero que meu corpo seja muito magro | I want my body to appear very skinny. | I want my body to look very thin. | Eu quero que meu corpo pareça muito magro. |
| I think a lot about looking muscular. | Eu penso muito sobre parecer forte. | I think a lot about seeming masculine. | I think about looking muscular a lot. | Eu gasto muito tempo pensando em ser musculoso. |
| I think a lot about looking thin. | Eu penso muito sobre parecer magro. | I think a lot about appearing skinny. | I think about looking thin a lot. | Eu gasto muito tempo pensando em ser magro. |
| I want my body to look muscular. | Eu quero que meu corpo se pareça forte. | I want my body to appear muscular | I want my body to look muscular. | Eu quero que meu corpo pareça musculoso. |
| I don't really think much about my appearance. | Eu não penso muito sobre meu corpo. | I really do not think about my appearance very much. | I honestly don't think much about my appearance. | Eu gasto muito tempo pensando em minha aparência. |
| I don't think much about how I look. | Eu não penso muito sobre meu corpo. | I really do not think about my appearance very much. | I honestly don't think much about my appearance. | *^a^* |
| I would like to have a body that looks very muscular. | Eu gostaria de ter um corpo muito forte. | I would like to have a very muscular body. | I would like to have a very muscular body. | Sinto que minha família me pressiona a melhorar a minha aparência. |
| I feel pressure from family members to look thinner. | Meus pais falam para que eu pareça mais magro. | I feel pressured by my family members to appear skinnier. | I feel pressure from my family to look thinner. | *^b^* |
| I feel pressure from family members to improve my appearance. | Meus pais falam para que eu melhore o meu corpo. | I feel pressured by my family members to improve my appearance. | I feel pressure from my family to improve my appearance. | *^b^* |
| Family members encourage me to get in better shape. | Meus pais falam para que eu fique em boa forma. | My family members encourage me to get in better shape. | My family encourages me to get in better shape. | Minha família me incentiva a melhorar minha forma física. |
| I feel pressure from family members to be more muscular. | Meus pais falam para que eu seja mais forte. | I feel pressured by my family members to be more muscular. | I feel pressured by my family to be more muscular. | Sinto que minha família me pressiona a ser mais musculoso. |
| Family members encourage me to increase the size or definition of my muscles. | Meus pais falam para eu aumentar o tamanho ou a definição dos meus músculos. | My family members encourage me to increase the size or the definition of my muscles. | My family encourages me to increase the size or definition of my muscles. | Minha família me incentiva a aumentar o tamanho de meus músculos. |
| I feel pressure from my peers to improve my appearance. | Meus amigos falam para que eu melhore a forma do meu corpo. | I feel pressured by my colleagues to improve my appearance. | I feel pressured by my colleagues to improve my appearance. | Sinto que meus amigos me pressionam a melhorar minha forma física. |
| I feel pressure from my peers to look in better shape. | Meus amigos falam para que eu fique em boa forma. | I feel pressured by my colleagues to be in better shape. | I feel pressured by my colleagues to be in better shape. | *^b^* |
| I feel pressure from my peers to be more muscular. | Meus amigos falam para que eu seja mais forte. | I feel pressured by my colleagues to stay more muscular. | I feel pressured by my colleagues to become more muscular. | Sinto que meus amigos me pressionam a ser mais musculoso. |
| My peers encourage me to increase the size or definition of my muscles. | Meus amigos falam para eu aumentar o tamanho ou a definição dos meus músculos. | My colleagues encourage me to increase the size or the definition of my muscles. | My colleagues encourage me to increase the size or definition of my muscles. | Meus amigos me incentivam a aumentar o tamanho de meus músculos. |
| I feel pressure from significant others to improve my appearance. | Pessoas próximas falam para que eu melhore a forma do meu corpo. | I feel pressured by the people close to me to improve my appearance. | I feel pressured by people around me to improve my appearance. | Sinto que pessoas próximas me pressionam a melhorar minha aparência. |
| I feel pressure from significant others to look in better shape. | Pessoas próximas falam para que eu fique em boa forma. | I feel pressured by the people close to me to stay in better shape. | I feel pressured by people around me to get in better shape. | Sinto que pessoas próximas me pressionam a melhorar minha forma física. |
| I get pressure from significant others to decrease my level of body fat. | Pessoas próximas falam para eu diminuir a gordura do meu corpo. | I feel pressured by the people close to me to lower my body fat level. | I feel pressured by people around me to lower my percentage of body fat. | Sinto que pessoas próximas me pressionam a emagrecer. |
| I feel pressure from significant others to be more muscular. | Pessoas próximas falam que eu seja mais forte. | I feel pressured by the people close to me to be more muscular. | I feel pressured by people around me to be more muscular. | Sinto que pessoas próximas me pressionam a ser mais musculoso. |
| I feel pressure from significant others to increase the size or definition of my muscles. | Pessoas próximas falam para eu aumentar o tamanho ou a definição dos meus músculos. | I feel pressured by the people close to me to increase the size or the definition of my muscles. | I feel pressured by people around me to increase the size or definition of my muscles. | Sinto que pessoas próximas me pressionam a aumentar o tamanho de meus músculos. |
| I feel pressure from the media to look in better shape. | A televisão e a internet me influenciam para que eu fique em boa forma. | I feel pressured by the media to be in better shape. | I feel pressured by the media to be in better shape. | Sinto que a TV e a Internet me pressionam a melhorar minha forma física. |
| I feel pressure from the media to look thinner. | A televisão e a internet me influenciam para que eu pareça mais magro. | I feel pressured by the media to appear skinnier. | I feel pressured by the media to look thinner. | Sinto que a TV e a Internet me pressionam a parecer mais magro. |
| I feel pressure from the media to improve my appearance. | A televisão e a internet me influenciam para que eu melhore a forma do meu corpo. | I feel pressured by the media to improve my appearance. | I feel pressured by the media improve my appearance. | Sinto que a TV e a Internet me pressionam a melhorar minha aparência. |
| I feel pressure from the media to decrease my level of body fat. | A televisão e a internet me influenciam para diminuir a gordura do meu corpo. | I feel pressured by the media to lower my body fat level. | I feel pressured by the media to lower my percentage of body fat. | Sinto que a TV e a Internet me pressionam a emagrecer. |
| I feel pressure from the media to be more muscular | A televisão e a internet me influenciam para que eu seja mais forte. | I feel pressured by the media to be more muscular. | I feel pressured by the media to be more muscular. | Sinto que a TV e a Internet me pressionam a ser mais musculoso. |
| I feel pressure from the media to increase the size or definition of my muscles. | A televisão e a internet me influenciam para eu aumentar o tamanho ou a definição dos meus músculos. | I feel pressured by the media to increase the size or the definition of my muscles. | I feel pressured by the media to increase the size or definition of my muscles. | Sinto que a TV e a Internet me pressionam a aumentar o tamanho de meus músculos. |

***Note: ^a^=Item deleted at Stage 3 (experts committee); ^b^=Item deleted at EFA***
