# Supplementary material for: Translation, Adaptation and Psychometric Properties of SATAQ-4R for Brazilian Children
Source: Psicol Reflex Crit. 2020 Jul 2;33:12. doi: 10.1186/s41155-020-00149-6 (PMC7332665; doi:10.1186/s41155-020-00149-6)
Supplement: Supplementary file 2 — Additional file 2. Questionário de Atitudes Socioculturais em Relação à Aparência – 4 R – MASCULINA and Questionário de Atitudes Socioculturais em Relação à Aparência – 4 R – FEMININA. [file 41155_2020_149_MOESM2_ESM.docx]

**Questionário de Atitudes Socioculturais em Relação à Aparência – 4 R - MASCULINA**

| Por favor, leia cada um dos itens e marque a mãozinha que melhor representa a sua opinião.  👎= **Não**; 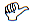= **Mais ou menos**; 👍= **Sim** |
| --- |

| 1. Para mim, é importante parecer musculoso. | 👎 | 👍 | 👍 |
| --- | --- | --- | --- |
| 1. Eu quero que meu corpo pareça muito magro. | 👎 | 👍 | 👍 |
| 1. Eu gasto muito tempo pensando em ser musculoso. | 👎 | 👍 | 👍 |
| 1. Eu gasto muito tempo pensando em ser magro. | 👎 | 👍 | 👍 |
| 1. Eu quero que meu corpo pareça musculoso. | 👎 | 👍 | 👍 |
| 1. Eu gasto muito tempo pensando em minha aparência. | 👎 | 👍 | 👍 |
| 1. Eu gostaria de ter um corpo muito musculoso. | 👎 | 👍 | 👍 |

Agora, responda às questões seguintes pensando na sua família: pais, irmãos, irmãs, avós, tios, primos.

| 1. Sinto que minha família me pressiona a melhorar a minha aparência. | 👎 | 👍 | 👍 |
| --- | --- | --- | --- |
| 1. Minha família me incentiva a melhorar minha forma física. | 👎 | 👍 | 👍 |
| 1. Sinto que minha família me pressiona a ser mais musculoso. | 👎 | 👍 | 👍 |
| 1. Minha família me incentiva a aumentar o tamanho de meus músculos. | 👎 | 👍 | 👍 |

Agora, responda às questões seguintes pensando nos seus amigos próximos, colegas de turma ou em outras crianças da sua idade.

| 1. Sinto que meus amigos me pressionam a melhorar minha forma física. | 👎 | 👍 | 👍 |
| --- | --- | --- | --- |
| 1. Sinto que meus amigos me pressionam a ser mais musculoso. | 👎 | 👍 | 👍 |
| 1. Meus amigos me incentivam a aumentar o tamanho de meus músculos. | 👎 | 👍 | 👍 |

Agora, responda às questões seguintes pensando em outras pessoas próximas a você, como seus professores, treinadores, vizinhos.

| 1. Sinto que pessoas próximas me pressionam a melhorar minha aparência. | 👎 | 👍 | 👍 |
| --- | --- | --- | --- |
| 1. Sinto que pessoas próximas me pressionam a melhorar minha forma física. | 👎 | 👍 | 👍 |
| 1. Sinto que pessoas próximas me pressionam a emagrecer. | 👎 | 👍 | 👍 |
| 1. Sinto que pessoas próximas me pressionam a ser mais musculoso. | 👎 | 👍 | 👍 |
| 1. Sinto que pessoas próximas me pressionam a aumentar o tamanho de meus músculos. | 👎 | 👍 | 👍 |

Agora, responda às questões seguintes pensando no que você assiste na TV, nos filmes, no que acessa na Internet e no que lê nas revistas.

| 1. Sinto que a TV e a Internet me pressionam a melhorar minha forma física. | 👎 | 👍 | 👍 |
| --- | --- | --- | --- |
| 1. Sinto que a TV e a Internet me pressionam a parecer mais magro. | 👎 | 👍 | 👍 |
| 1. Sinto que a TV e a Internet me pressionam a melhorar minha aparência. | 👎 | 👍 | 👍 |
| 1. Sinto que a TV e a Internet me pressionam a emagrecer. | 👎 | 👍 | 👍 |
| 1. Sinto que a TV e a Internet me pressionam a ser mais musculoso. | 👎 | 👍 | 👍 |
| 1. Sinto que a TV e a Internet me pressionam a aumentar o tamanho de meus músculos. | 👎 | 👍 | 👍 |

**Questionário de Atitudes Socioculturais em Relação à Aparência – 4R - FEMININA**

| **Por favor, leia cada um dos itens e marque a mãozinha que melhor representa a sua opinião.**  👎= **Não**; 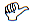= **Mais ou menos**; 👍= **Sim** |
| --- |

| 1. Para mim, é importante parecer musculosa. | 👎 | 👍 | 👍 |
| --- | --- | --- | --- |
| 1. Eu quero que meu corpo pareça muito magro. | 👎 | 👍 | 👍 |
| 1. Eu gasto muito tempo pensando em ser musculosa. | 👎 | 👍 | 👍 |
| 1. Eu gasto muito tempo pensando em ser magra. | 👎 | 👍 | 👍 |
| 1. Eu quero que meu corpo pareça musculoso. | 👎 | 👍 | 👍 |
| 1. Eu gasto muito tempo pensando em emagrecer. | 👎 | 👍 | 👍 |
| 1. Eu gostaria de ter um corpo muito musculoso. | 👎 | 👍 | 👍 |

Agora, responda às questões seguintes pensando na sua família: pais, irmãos, irmãs, avós, tios, primos.

| 1. Sinto que minha família me pressiona a ser mais magra. | 👎 | 👍 | 👍 |
| --- | --- | --- | --- |
| 1. Sinto que minha família me pressiona a melhorar minha aparência. | 👎 | 👍 | 👍 |
| 1. Minha família me incentiva a emagrecer. | 👎 | 👍 | 👍 |

Agora, responda às questões seguintes pensando em seus amigos próximos, colegas de turma ou em outras crianças da sua idade.

| 1. Meus amigos me incentivam a ficar mais magra. | 👎 | 👍 | 👍 |
| --- | --- | --- | --- |
| 1. Sinto que meus amigos me pressionam a melhorar minha aparência. | 👎 | 👍 | 👍 |
| 1. Sinto que meus amigos me pressionam a melhorar minha forma física. | 👎 | 👍 | 👍 |
| 1. Meus amigos me pressionam a emagrecer. | 👎 | 👍 | 👍 |

Agora, responda às questões seguintes pensando em outras pessoas próximas a você, como seus professores, treinadores, vizinhos.

| 1. Pessoas próximas me incentivam a ficar mais magra. | 👎 | 👍 | 👍 |
| --- | --- | --- | --- |
| 1. Sinto que pessoas próximas me pressionam a melhorar minha aparência. | 👎 | 👍 | 👍 |
| 1. Sinto que pessoas próximas me pressionam a melhorar minha forma física. | 👎 | 👍 | 👍 |
| 1. Sinto que pessoas próximas me pressionam a emagrecer. | 👎 | 👍 | 👍 |

Agora, responda às questões seguintes pensando no que você assiste na TV, nos filmes, no que acessa na Internet e lê nas revistas.

| 1. Sinto que a TV e a Internet me pressionam a melhorar minha forma física. | 👎 | 👍 | 👍 |
| --- | --- | --- | --- |
| 1. Sinto que a TV e a Internet me pressionam a parecer mais magra. | 👎 | 👍 | 👍 |
| 1. Sinto que a TV e a Internet me pressionam a melhorar minha aparência. | 👎 | 👍 | 👍 |
| 1. Sinto que a TV e a Internet me pressionam a emagrecer. | 👎 | 👍 | 👍 |
